# Ophiopogonin D of *Ophiopogon japonicus* ameliorates renal function by suppressing oxidative stress and inflammatory response in streptozotocin-induced diabetic nephropathy rats

**DOI:** 10.1590/1414-431X20209628

**Published:** 2020-06-03

**Authors:** Yanhong Qiao, Haiyan Jiao, Feng Wang, Huimin Niu

**Affiliations:** Nephrology Department, Heping Hospital affiliated to Changzhi Medical College, Changzhi, Shanxi, China

**Keywords:** Hyperglycemia, Pro-inflammatory cytokines, Oxidative damage, Renal dysfunction

## Abstract

Ophiopogonin D (OP-D) is the principal pharmacologically active ingredient from *Ophiopogon japonicas*, which has been demonstrated to have numerous pharmacological activities. However, its protective effect against renal damage in streptozotocin (STZ)-induced diabetic nephropathy (DN) rats remains unclear. The present study was performed to investigate the protective effect of OP-D in the STZ-induced DN rat model. DN rats showed renal dysfunction, as evidenced by decreased serum albumin and creatinine clearance, along with increases in serum creatinine, blood urea nitrogen, TGF-β1, and kidney hypertrophy, and these were reversed by OP-D. In addition, STZ induced oxidative damage and inflammatory response in diabetic kidney tissue. These abnormalities were reversed by OP-D treatment. The findings obtained in the present study indicated that OP-D might possess the potential to be a therapeutic agent against DN via inhibiting renal inflammation and oxidative stress.

## Introduction

Diabetes mellitus is a chronic metabolic disorder, primarily characterized by hyperglycemia arising from insulin insensitivity, damaged secretion of insulin, and inflammatory response (1). Diabetes mellitus has been considered the third most serious disease for humans. Long-term hyperglycemia is the leading cause of various diabetic complications, such as neuropathy and nephropathy (2). Diabetic nephropathy (DN) is a progressive diabetic complication, which might initially lead to nephrotic syndrome and eventually develop into chronic kidney disease and end-stage renal disease (3).

Although the full molecular mechanisms involved in the pathogenesis and development of DN is still insufficiently understood, increasing evidence indicates that inflammation and oxidative stress induced by hyperglycemia play a vital role in the progression of DN (4). In diabetes, hyperglycemia usually promotes oxidative stress either by altering the redox balance or by excessive generation of reactive oxygen species (5). Furthermore, increased oxidative stress and excessive production of reactive oxygen species result in enzyme inactivation, alteration in antioxidant gene expression, and cell membrane injury (6). The excessive generation of reactive oxygen species causes renal failure, resulting in increased albumin excretion and glomerular barrier dysfunction (7). On the other hand, the development and progression of DN is related to increased serum levels of inflammatory cytokines and obvious inflammatory cell infiltration, such as interleukin 6 (IL-6), interleukin-1β (IL-1β), tumor necrosis factor α (TNF-α), and vascular cell adhesion molecule-1 (VCAM-1) (8). Thus, it is important to alleviate diabetic pathophysiology by multi-targeted therapeutic agents. Therefore, counteracting inflammation and oxidative stress might effectively suppress renal failure and prevent or alleviate the progression of DN (9). However, currently, chemically synthesized drugs are unable to effectively slow the progression of DN, thus, the need for developing more effective and novel therapeutic agents to treat DN is urgent.

In recent years, the confidence of DN patients on medicinal plant-based therapy for DN has been gradually increasing. *Radix Ophiopogon japonicas*, a traditional Chinese herbal medicine, has been used to treat diabetes mellitus and research has demonstrated its beneficial effects on DN in experimental animals (10–12). Ophiopogonin D (OP-D) is a steroidal glycoside and the chief pharmacologically active component derived from *Radix Ophiopogon japonicas*, which has been demonstrated to possess numerous pharmacological activities, such as anti-oxidative, inhibition of venous thrombosis, and anti-inflammatory activities (13–15). A previous study has indicated that the possible anti-inflammatory mechanism of OP-D is related to the suppression of the NF-κB signaling pathway (16). It was also reported that OP-D suppresses Ang II-induced activation of pro-inflammatory cytokines (IL-6, VCAM-1, and TNF-α) and NF-κB nuclear translocation (17). These activities of OP-D indicate its potential prevention effects in DN. However, there is no research indicating the use of OP-D as an anti-DN agent and the underlying molecular mechanism remains unclear. Thus, the purpose of present study was to explore the effect of OP-D on DN in the streptozotocin (STZ)-induced diabetic rat model and investigate the possible underlying action mechanism of OP-D on DN.

## Material and Methods

### Drugs and reagents

OP-D (purity ≥98%, HLPC) was obtained from Sichuan Weikeqi Biological Techology Co. (China). STZ was obtained from Sigma-Aldrich (USA) and gliclazide (Glic, 99.2%) was obtained from the Servier (Tianjin) Pharmaceutical Co. (China).

### Experimental animals

Male Sprague Dawley rats (weight 200-220 g) were purchased from Hunan University Laboratory Animal Center (China). All animals were housed under constant humidity (50-60%), temperature (22±2°C), and a 12-h light/dark cycle. All rats were fed a standard diet and allowed free access to water. The animal study protocol was carried out according to the National Institutes of Health guidelines and approved by the Animal Use Ethics Committee for the care and use of experimental animals (Heping Hospital affiliated to Changzhi Medical College).

### Induction of experimental diabetes

After a week of accommodation, the diabetes model was induced by a single intraperitoneal (*ip*) injection of freshly prepared STZ (65 mg/kg, dissolved in 0.1 M citrate buffer, pH 4.5) after overnight fasting. The control group of rats received an equal volume of citrate buffer (0.1 M, pH 4.5) (18). About 72 h after injection, the success of diabetes induction was confirmed by assaying the fasting blood glucose (FBG) levels with a reagent kit (Nanjing Jiancheng Bioengineering Institute, China). The rats with an FBG level higher than 16.7 mM were considered diabetic and used for further pharmacological assessments (19).

### Experimental protocol

The diabetic rats were randomly divided into five groups (12 rats per group) as follows: NC: Normal control group rats treated with 0.1% of sodium carboxymethyl cellulose (CMC-Na); DN: diabetic rats treated with an equal volume of 0.1% CMC-Na solution without drug treatment; Glic: diabetic rats treated with 5 mg/kg gliclazide daily by oral gavage for 12 weeks (20); OP-D-L: diabetic rats received 2.5 mg/kg body weight OP-D by oral gavage daily for 12 weeks (16); OP-D-M: diabetic rats received 5 mg/kg body weight OP-D by oral gavage daily for 12 weeks (16); OP-D-H: diabetic rats received 10 mg/kg body weight OP-D by oral gavage daily for 12 weeks (16).

All drugs were suspended in 0.1% CMC-Na solution. The level of FBG was assayed every four weeks.

### Sample preparation

One day before the last experiment, 24-h urine was collected and the albumin levels were measured after the urine was pooled. At the end of experiment, blood samples were obtained from the retro-orbital sinus plexus, the serum was separated by centrifugation at 5000 *g* for 10 min at 3°C, and the supernatant was collected and stored at -80°C for further analysis by biochemical assays. The animals were then anesthetized and executed by cervical dislocation. Kidney tissues were immediately excised, cleaned, and weighed. Then, the kidney tissues were homogenized in ice-cold normal saline at a ratio of 1:9 (w/v). The homogenate was centrifuged at 12,000 *g* for 10 min at 3°C and the supernatant was collected and stored at -80°C until further analysis.

Kidney hypertrophy was calculated as follows: Kidney hypertrophy = kidney weight (g) / body weight (g).

### Measurement of fasting blood glucose (FBG) and blood HbA1c

After animals were fasted for 12 h, FBG levels were determined at 0, 4, 8, and 12 weeks using commercially available kits (Nanjing Jiancheng Bioengineering Institute) according to the manufacturer's instructions. Blood HbA1c% was measured using commercial kits purchased from Nanjing Jiancheng Bioengineering Institute in accordance with the manufacturer's protocol.

### Evaluation of renal function

Serum creatinine, blood urea nitrogen, and serum albumin were assayed using a biochemical analyzer (INFORS-YSI 2900, Switzerland). The plasma TGF-β1 levels were assayed using a commercially available ELISA kit (Nanjing Jiancheng Bioengineering Institute) according to the manufacturer's protocol.

### Histological assessment

The renal tissue was preserved with 10% formalin, dehydrated, and embedded in paraffin. Then, renal tissue was cut into 5-mm sections and renal fibrosis was evaluated by Masson trichrome staining. The sections were stained for 8 min using hematoxylin solution after the renal tissue was deparaffinized and rehydrated. After washing with tap water for 8 min, the sections were stained for 5 min using ponceau-fuchsin acid solution. The sections were impregnated in aniline blue solution for 5 min and then differentiated for 5 min with phosphomolybdic-phosphotungstic acid. Finally, the sections were differentiated for 2 min with 0.2% acetic acid, followed by dehydration and washing. The pathological microscopic alterations were observed under light microscopy at 200× magnification.

### Measurement of pro-inflammatory cytokines

IL-6 and IL-1β levels in kidney homogenate were assayed by ELISA kits provided by Nanjing Jiancheng Bioengineering Institute following the manufacturer's protocol. The protein content of kidney homogenate was assayed according to a previously reported method (21).

### Measurement of malondialdehyde and antioxidant enzyme activity

The activities of superoxide dismutase (SOD), glutathione peroxidase (GSH), and catalase (CAT) and the levels of malondialdehyde (MDA) in the kidney homogenate were measured using commercial kits (Nanjing Jiancheng Bioengineering Institute) according to the manufacturer's instruction.

### Western blotting

Kidney tissues were lysed in RIPA lysis buffer (Pierce; USA) and total protein of the lysates was measured using a BCA assay kit (Nanjing Jiancheng Bioengineering Institute). Lysates were separated by 12% SDS-polyacrylamide gels and transferred to a PVDF membrane (Millipore, USA). The membranes were blocked with 5% nonfat dried milk in TBST buffer for 1 h at room temperature. Blotted membranes were incubated with the following primary antibodies overnight at 4°C: anti-IL-6 (1:200; Invitrogen, USA), anti-TNF-α (1:200; Invitrogen), anti-NF-κB (1:200; Invitrogen), and GAPDH (1:200; Invitrogen). Then, the membranes were washed with TBST and incubated with HRP-labeled goat anti-mouse IgG antibody for 1 h at room temperature. Visualization was carried out with the Pierce ECL Western blotting substrate. The relative protein levels were assayed using Gel-Pro 4.0 software (Media Cybernetics, Inc., USA) and normalized to GAPDH.

### Statistical analysis

Data are reported as means±SE. Statistical analysis was carried out using SPSS (version 18.0, USA). The differences between all groups were compared using one-way ANOVA followed by Tukey's *post hoc* analysis. Comparison between the two groups was conducted using Student's *t*-test. P<0.05 was considered statistically significant.

## Results

### Effect of OP-D on renal hypertrophy

The renal size of the DN rats was increased compared to the control rats, indicating that renal hypertrophy occurred in the DN rats. In contrast, the renal hypertrophy was decreased in both the OP-D and Glic groups compared to that in the DN group (P<0.05 or P<0.01), indicating that OP-D exerted a protective effect by ameliorating renal hypertrophy in the DN group (Table 1).

**Table 1:** Effect of high, medium, and low (H, M, L) ophiopogonin D (OP-D) on renal hypertrophy in streptozotocin-induced diabetic nephropathy (DN) rats.

	Control	DN	Glic	OP-D-H	OP-D-M	OP-D-L
Body weight (g)	312.56±11.79	173.56±7.45^##^	314.39±10.52**	311.47±8.98**	253.79±6.80**	208.42±9.45*
Kidney weight (g)	1.23±0.02	1.59±0.11^##^	1.22±0.04**	1.23±0.04**	1.34±0.03*	1.43±0.03
Kidney hypertrophy (g)	0.39±0.02	0.92±0.08^##^	0.39±0.02**	0.40±0.02**	0.53±0.02**	0.68±0.03*

Data are reported as means±SE (n=12). ^##^P<0.01 *vs* control group, *P<0.05 and **P<0.01 *vs* DN group (ANOVA followed by Tukey's *post hoc* analysis). Glic: Gliclazide.

### OP-D ameliorated hyperglycemia in DN rats

**Figure 1:**
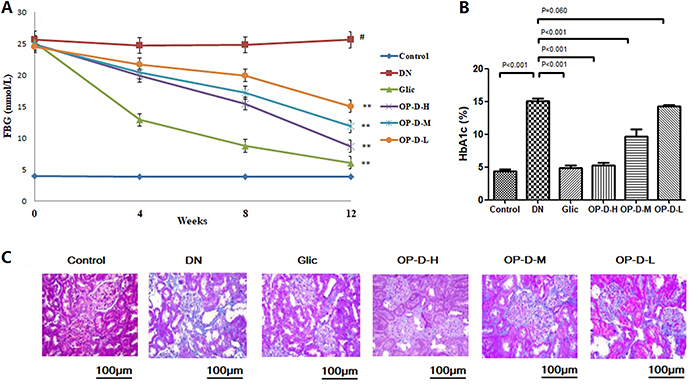
High, medium, and low (H, M, L) ophiopogonin D (OP-D) attenuated hyperglycemia in streptozotocin-evoked diabetic nephropathy (DN) rats. Treatment with OP-D decreased fasting blood glucose (FBG) levels (**A**) and HbA1c% (**B**). **C**, Masson staining for renal fibrosis in kidney of rats (200× magnification, bar: 100 µm). Data are reported as means±SE (n=12). ^#^P<0.01 *vs* control group, **P<0.01 *vs* DN group (ANOVA followed by Tukey's *post hoc* analysis). Glic: Gliclazide.

The DN group showed an increase in the FBG levels compared to the control group. Hyperglycemia was observed in the DN group during the study period compared with the control group. Treatment with OP-D or Glic reduced FBG levels in DN rats (P<0.05 or P<0.01, Figure 1A). The anti-hyperglycemic effect of OP-D was confirmed by the values of HbA1c%. DN rats showed a significant increase (P<0.01) in HbA1c% compared to control rats (Figure 1B). Treatment with OP-D-H or OP-D-M significantly reduced HbA1c% levels in DN rats (P<0.05 or P<0.01).

### Effect of OP-D on histopathological alterations

Renal interstitium of diabetic rats showed mesangial hyperplasia and abnormal glomerular architecture. However, treatment with OP-D or Glic improved the morphological and structural characteristics of the glomerulus (Figure 1C).

### Effect of OP-D on serum biochemical parameters

**Figure 2:**
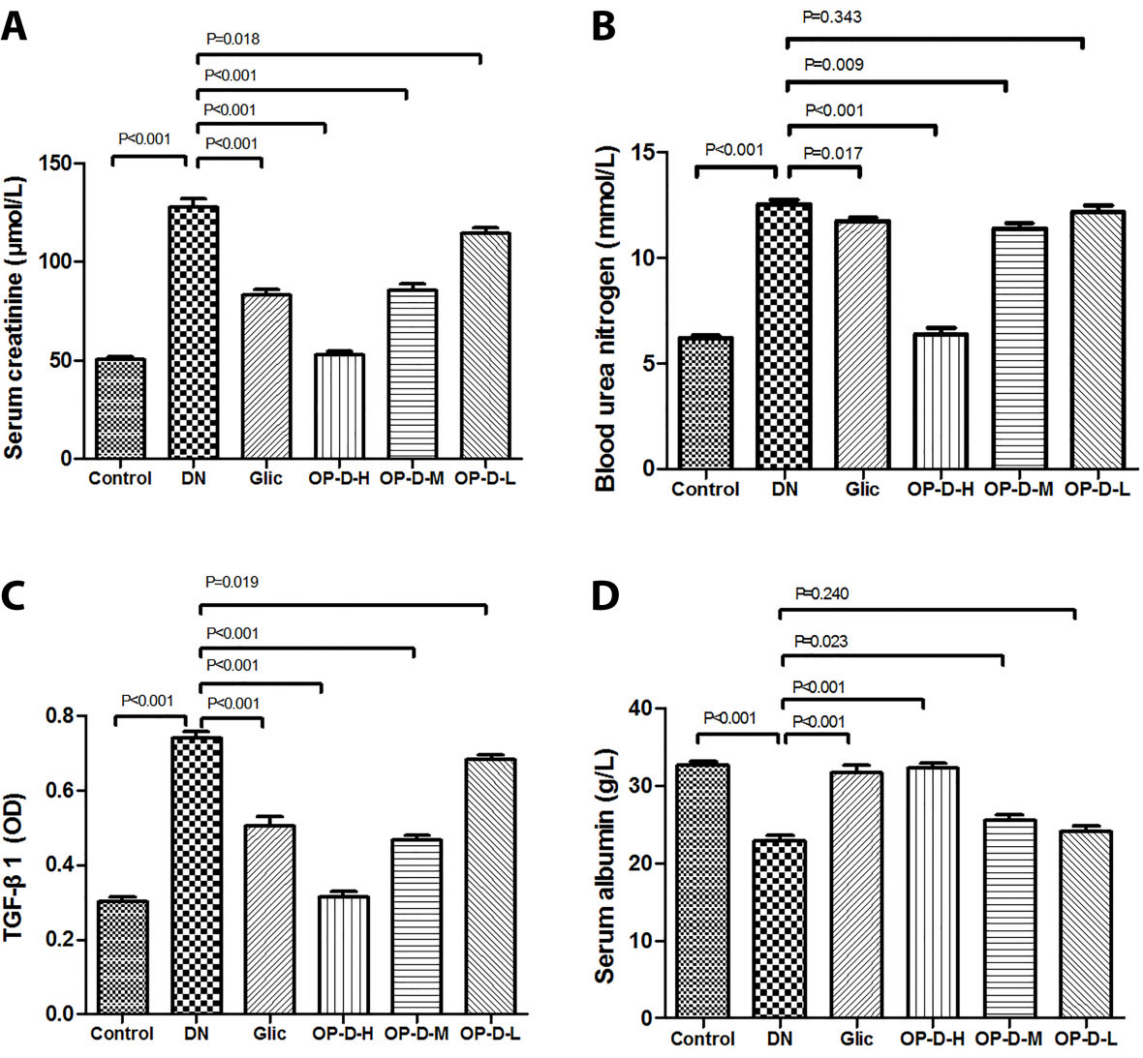
Effect of high, medium, and low (H, M, L) ophiopogonin D (OP-D) on serum biochemical parameters in streptozotocin-evoked diabetic nephropathy (DN) rats: serum creatinine (**A**), blood urea nitrogen (**B**), TGF-β1 (**C**), and serum albumin (**D**). Data are reported as means±SE (n=12) (ANOVA followed by Tukey's *post hoc* analysis). Glic: Gliclazide.

DN rats showed increased serum creatinine and blood urea nitrogen levels, as well as a decline in serum albumin levels compared to control rats (P<0.01). However, treatment with OP-D-H or OP-D-M decreased the serum creatinine and blood urea nitrogen levels, as well as increased serum albumin levels in DN rats (P<0.05 or P<0.01, Figure 2A, B, and D).

### Effect of OP-D on plasma TGF-β1 level

DN rats showed increased plasma TGF-β1 level compared to control rats (P<0.01). However, treatment with OP-D-H or OP-D-M reversed these alterations in the DN rats (P<0.05 or P<0.01, Figure 2C).

### Effect of OP-D on urine parameters

**Figure 3:**
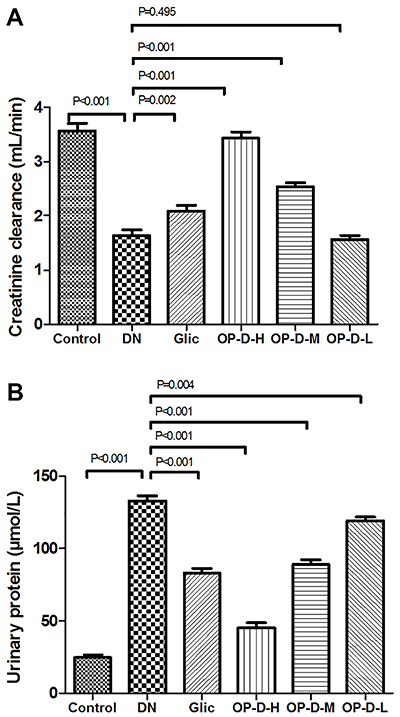
Effect of high, medium, and low (H, M, L) ophiopogonin D (OP-D) on urine parameters in streptozotocin-evoked diabetic nephropathy (DN) rats. Creatinine clearance (**A**) and urinary protein (**B**) are shown. Data are reported as means±SE (n=12) (ANOVA followed by Tukey's *post hoc* analysis). Glic: Gliclazide.

DN rats showed a significant decrease in the creatinine clearance levels, as well as a significant increase in the urinary protein levels compared to control rats (P<0.01). However, treatment with OP-D-H or OP-D-M decreased the urinary protein levels, as well as increased the creatinine clearance levels in the DN rats (P<0.05 or P<0.01, Figure 3).

### Effect of OP-D on renal antioxidant status and oxidative stress

**Figure 4:**
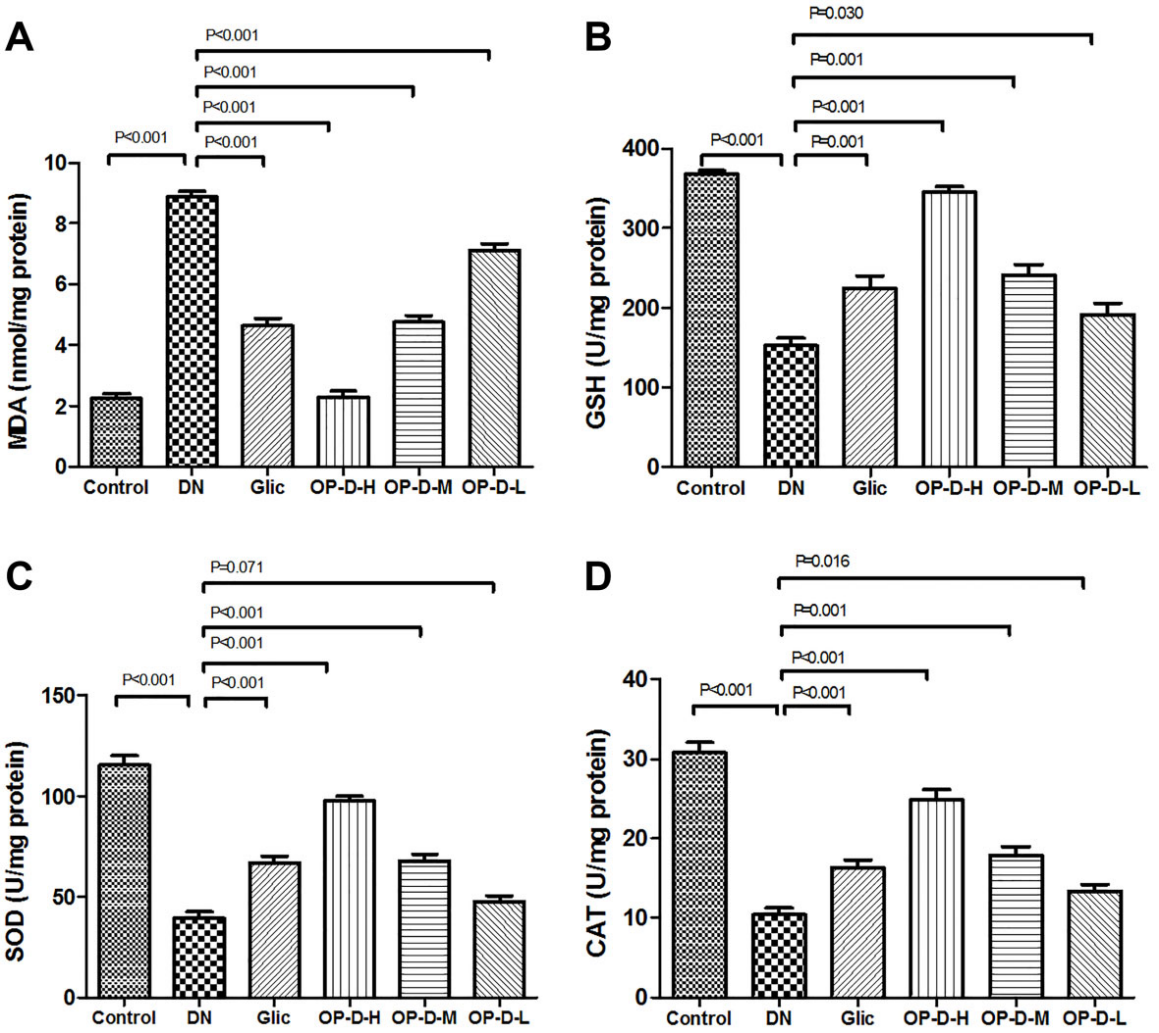
Effect of high, medium, and low (H, M, L) ophiopogonin D (OP-D) on kidney oxidative stress parameters in streptozotocin-evoked diabetic nephropathy (DN) rats. Malondialdehyde (MDA) (**A**), glutathione peroxidase (GSH) (**B**), superoxide dismutase (SOD) (**C**), and catalase (CAT) are shown (**D**). Data are reported as means±SE (n=12) (ANOVA followed by Tukey's *post hoc* analysis). Glic: Gliclazide.

DN rats showed an increase in kidney MDA levels, as well as a decline in kidney antioxidant enzymes levels (GSH, SOD, and CAT) compared to control rats (P<0.01). However, treatment with OP-D-H or OP-D-M decreased MDA levels, while restoring the activities of GSH, SOD, and CAT in the kidney tissue (P<0.05 or P<0.01, Figure 4).

### OP-D alleviated inflammation in the kidney of DN rats

**Figure 5:**
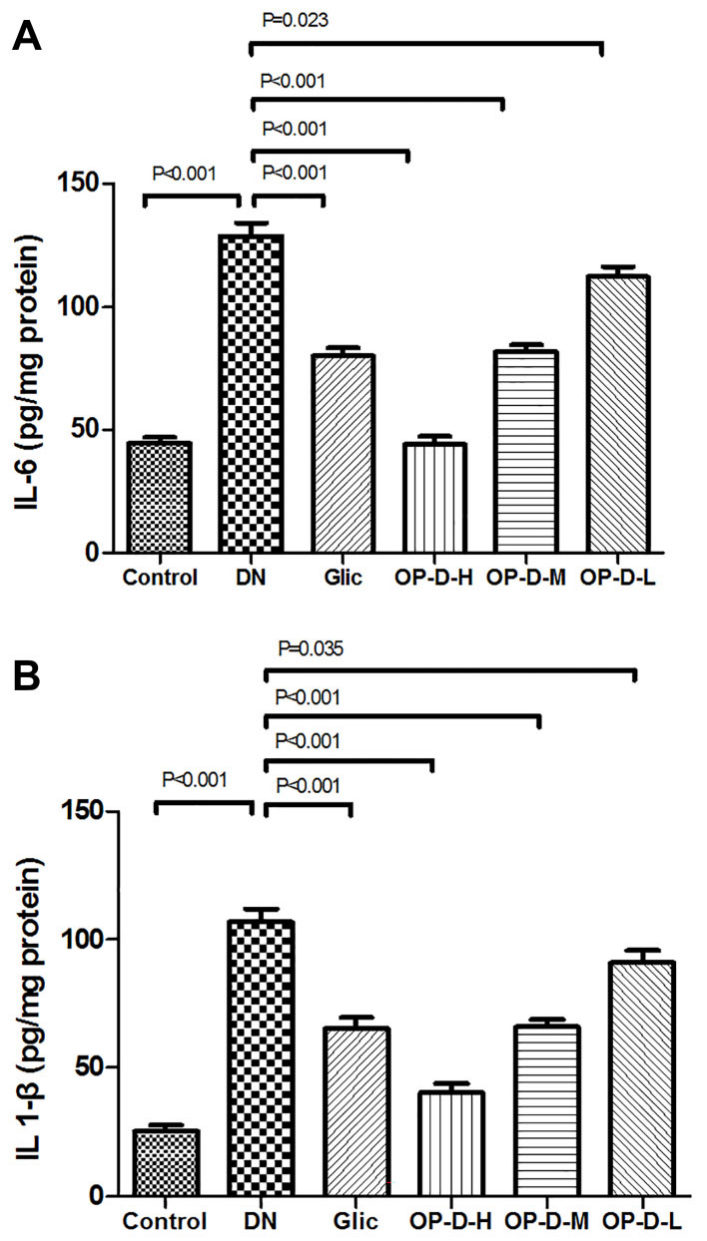
Effect of high, medium, and low (H, M, L) ophiopogonin D (OP-D) on kidney pro-inflammatory factors in streptozotocin-evoked diabetic nephropathy (DN) rats. Interleukin (IL)-6 (**A**) and IL-1β (**B**) are shown. Data are reported as means±SE (n=12) (ANOVA followed by Tukey's *post hoc* analysis). Glic: Gliclazide.

DN rats showed increased kidney IL-6 and IL-1β levels compared to control rats (P<0.01). However, treatment of DN rats with OP-D decreased the levels of pro-inflammatory markers compared to DN rats (P<0.05 or P<0.01, Figure 5).

### Effect of OP-D on inflammatory markers protein expression

**Figure 6:**
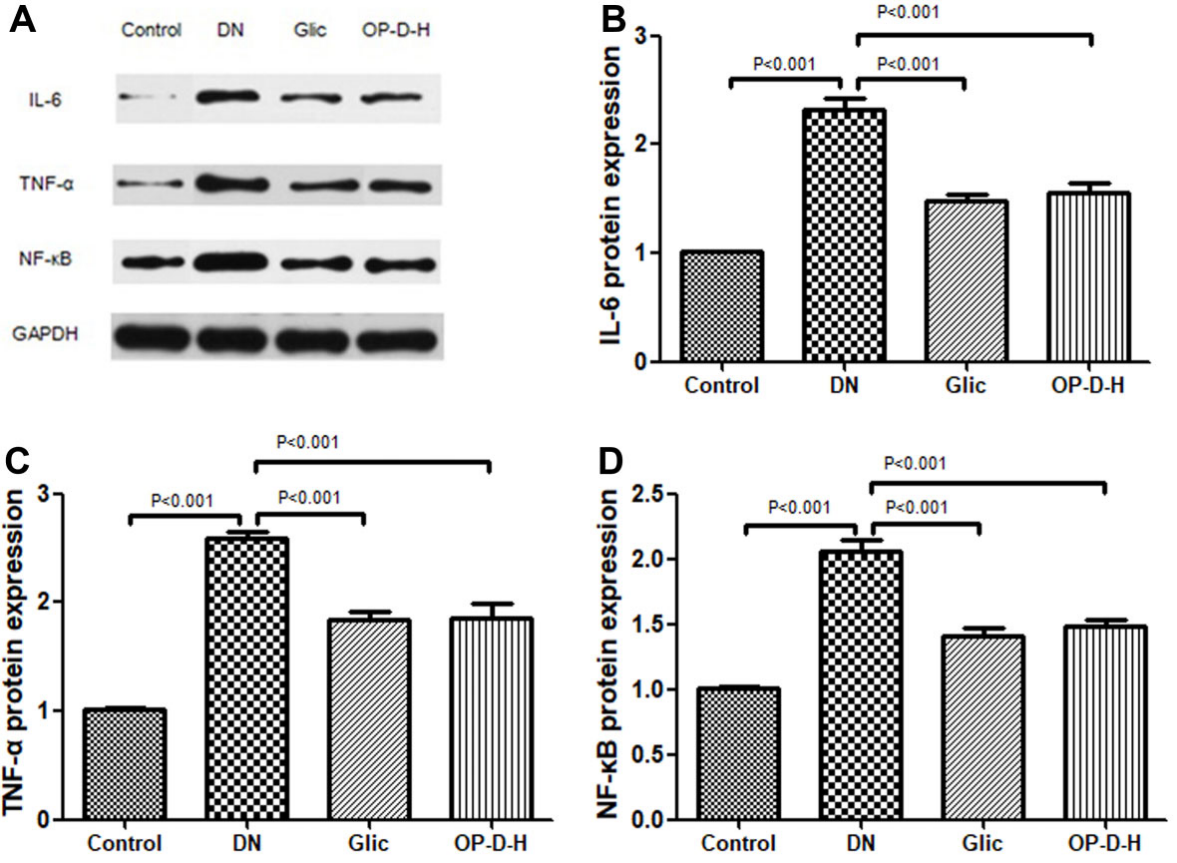
**A**, Western blot analysis. Effect of high ophiopogonin D (OP-D-H) on protein expression of interleukin (IL)-6 (**B**), tumor necrosis factor α (TNF-α) (**C**), and nuclear factor (NF)-κB (**D**). Data are reported as mean±SEM (n=8) (ANOVA followed by Tukey's *post hoc* analysis). Glic: Gliclazide.

Western blot analysis indicated that the protein expression of IL-6, TNF-α, and NF-κB in DN rats was significantly up-regulated compared to control rats (P<0.001). However, treatment with OP-D-H or Glic decreased the expression levels of IL-6, TNF-α, and NF-κB in the DN rats (P<0.001, Figure 6).

## Discussion

DN is a chronic metabolic disorder of the kidney, which is characterized by renal glomerular capillary damage and microvasculature disruption (22). Previous research indicates that diabetes-evoked metabolites, such as inflammatory markers, glucose, oxidative stress, and others, are involved in the progression and development of DN (9,23). High blood glucose levels induce oxidative stress by accelerating mitochondrial generation of reactive oxygen species (ROS) and glucose oxidation. Therefore, hyperglycemia results in DNA injury and aggravates apoptosis, which has been considered to be the major initiator of renal dysfunction related to diabetes (24). In the present study, we observed the typical symptoms in STZ-evoked DN rats including increased levels of HbA1c%, FBG, serum creatinine and blood urea nitrogen, renal hypertrophy, as well as kidney tissue oxidative stress and inflammatory damage. The protective effect of OP-D in STZ-induced DN rats was partly mediated by an anti-hyperglycemia effect and by the suppression of oxidative stress and inflammatory factors. STZ-induced renal injury is well demonstrated and is attributed to ROS production, which causes inflammatory outburst and oxidative stress (4,25). Therefore, natural medicines with anti-inflammatory and antioxidant effects may be potential therapeutic agents for prevention and treatment of DN.

Hyperglycemia-evoked oxidative stress is considered the crucial pathogenic factor for the progression of DN (26). Previous reports have demonstrated that STZ-induced DN is due to the production of lipid peroxidation products (27). In the present research, our findings indicated that STZ exposure induced the generation of MDA, decreased the activities of antioxidant enzymes, and caused oxidative injury in kidney tissue. We further showed that OP-D treatment for 12 weeks alleviated the antioxidant status of kidney tissue in DN rats. Our findings are in agreement with previous reports that revealed that OP-D exerts potent antioxidant effects (28).

Oxidative stress can lead to inflammatory response by several mechanisms, such as the activation of NF-κB (9). Pro-inflammatory factors play a vital role in the pathogenesis of DN (29). The levels of pro-inflammatory cytokines such as IL-6, TNF-α, and IL-1β are increased in DN (30). In addition, the release of pro-inflammatory factors in DN can accelerate the infiltration of macrophages, which in turn injures kidney tissue resulting in the high levels of urinary protein (31). Although the detailed pathway involving these pro-inflammatory cytokines in DN has not been fully understood, it has been implied that the activation of IL-6 leads to dysfunction in glomerular endothelial permeability (32). In addition, IL-1β, an autocrine growth factor, has been implicated in the progression and development of DN (33). Overexpression of TNF-α can accelerate the generation of ROS and induce oxidative damage in kidney tissue (31). In the present study, the DN rats showed increased levels of IL-6, TNF-α, and IL-1β, which were decreased by OP-D treatment, indicating that OP-D could protect the kidney via inhibiting the levels of pro-inflammatory cytokines. These findings are in agreement with a previous report, which revealed that OP-D exerts a potent anti-inflammatory effect *in vivo* (34). NF-κB is a ubiquitous transcription factor that takes part in regulating gene expressions that are implicated in inflammatory response and cell proliferation including chemokines, pro-inflammatory cytokines, and adhesion molecules (35). In this study, OP-D inhibited the activation of NF-κB in kidney tissue of DN rats, as shown by the down-regulation of NF-κB protein expression. OP-D exerted an anti-inflammatory effect by suppressing the NF-κB pathway, which was in agreement with a previous report (16). Our findings indicated that the suppression of renal inflammation by OP-D in DN rats was possibly via inhibition of the NF-κB activation. However, due to the limitation of the present research, the detailed mechanisms of OP-D need further investigation before it becomes a therapeutic agent for DN in clinical application.

TGF-β1 is responsible for alleviating glomerulosclerosis by regulating the protein synthesis of extracellular matrix in DN (25). Our results indicated that OP-D treatment decreased the level of TGF-β1 in DN rats, which might be related to the alleviation of inflammation and oxidative stress.

In summary, our findings indicated that the renoprotective effects of OP-D occurred by the improvement of renal function and glycemic control, which are involved in the suppression of oxidative stress and inflammatory response. Thus, we could conclude that OP-D could alleviate the development and progression of DN, and we anticipate that the present study will provide a theoretical basis for developing novel therapeutic methods for DN.
